# Chinese expert consensus on magnetic resonance-guided focused ultrasound surgery for painful bone metastases

**DOI:** 10.1186/s13244-026-02268-7

**Published:** 2026-04-07

**Authors:** Xuhui Fan, Na Tang, Jianjun Gu, Xiaorui Yin, Depeng Liu, Jiakang Shen, Junhai Zhang, Shengping Wang, Han Wang

**Affiliations:** 1 Department of Radiology, Shanghai General Hospital, Shanghai Jiao Tong University School of Medicine, Shanghai, China; 2 Department of Orthopedic Oncology, Shanghai Bone Tumor Institute, Shanghai General Hospital, Shanghai Jiao Tong University School of Medicine, Shanghai, China; 3 Department of Radiology, Huashan Hospital, Fudan University, Shanghai, China; 4 Department of Radiology, Shanghai Cancer Center, Fudan University, Shanghai, China; 5 Shanghai General Hospital Branch of National Center for Translational Medicine (Shanghai), Shanghai, China; 6 Jiading Branch of Shanghai General Hospital, Shanghai, China

**Keywords:** Magnetic resonance-guided focused ultrasound, ‌Metastatic bone pain, Expert consensus

## Abstract

**Abstract:**

Magnetic resonance-guided focused ultrasound surgery (MRgFUS) is an emerging noninvasive treatment for painful bone metastases. The therapeutic mechanism involves precise thermal ablation of periosteal nerve endings combined with controlled necrosis of tumor-involved soft tissue, achieving both immediate pain relief and local disease control. Compared to conventional treatments such as opioid therapy, radiation, or chemotherapy, MRgFUS offers distinct advantages, including non-addictiveness, rapid pain relief, minimal systemic side effects, and excellent patient tolerance. To standardize and optimize MRgFUS clinical practice, the Radiology Department Association of Hospitals of Shanghai and the Micro/Non-invasive Treatment Committee of the Chinese Research Hospital Association have jointly developed this expert consensus document through a structured face-to-face consensus meeting involving 29 experts, combined with anonymous voting. This consensus comprehensively outlines the clinical application of MRgFUS, detailing indications and contraindications, while establishing standardized protocols for preoperative multidisciplinary evaluation, intraoperative workflow (encompassing team responsibilities, technical specifications, treatment planning, and real-time thermal monitoring), postoperative care, adverse event management, and long-term follow-up to assess treatment efficacy and safety outcomes. This consensus further proposes future development strategies for MRgFUS, emphasizing the establishment of global case data registries for collaborative research and the application of AI technologies to identify optimal candidates, thereby standardizing and optimizing procedural efficiency to advance MRgFUS applications in painful bone metastases.

**Critical relevance statement:**

This expert consensus provides a detailed analysis of the advantages and target populations of MRgFUS, along with comprehensive technical considerations for the procedure.

**Key Points:**

- Magnetic resonance-guided focused ultrasound surgery (MRgFUS) requires expert consensus to standardize clinical application.
- MRgFUS is an effective alternative therapy‌, and this consensus detailed MRgFUS operational standards for painful bone metastases.
- This consensus enhances operator understanding of MRgFUS and facilitates its clinical adoption for painful bone metastases.

**Graphical Abstract:**

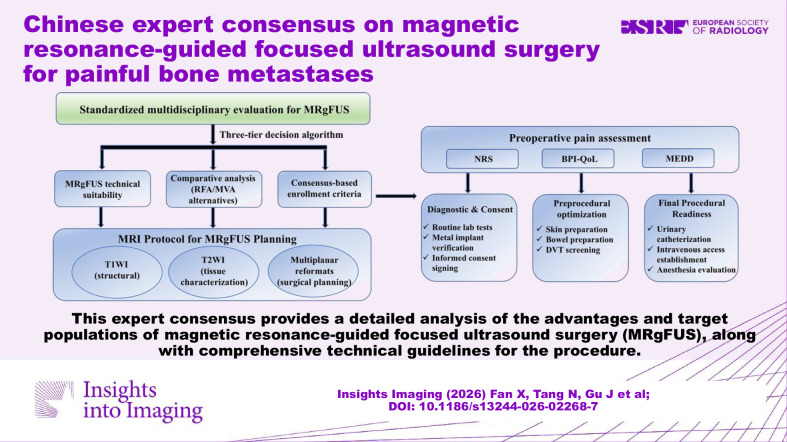

## Introduction

Painful bone metastases are defined as secondary bone lesions that cause persistent neuropathic pain. Autopsy studies revealed that bone micrometastases occur in 70–90% of patients with breast and prostate cancers [1–3]. The pain caused by bone metastasis progresses from intermittent to constant, with patients experiencing nocturnal exacerbations that disrupt sleep and daily function [4]. Current treatments for painful bone metastases include pharmacological options (opioids with variable efficacy and addiction risks, and bone-modifying agents like zoledronic acid associated with renal failure, and hypocalcemia), radiotherapy (50–58% pain relief but with 1–4 week latency and risk of radiation resistance), and surgery (primarily indicated for cases with spinal instability or pathological fractures requiring stabilization), driving the need for faster-acting, less invasive multimodal approaches [4–6].

Magnetic resonance-guided focused ultrasound (MRgFUS) is a cutting-edge, noninvasive treatment for painful bone metastases that combines real-time MRI guidance with precisely focused ultrasound energy to thermally destroy tumor tissue. The procedure works by delivering concentrated ultrasound waves to the metastatic bone lesion, generating localized heat (65–85 °C) that both ablates the tumor and disrupts pain-transmitting nerves in the surrounding periosteum [7, 8]. This dual mechanism provides rapid pain relief, often within 3 days, by directly targeting the source of pain while avoiding radiation exposure or surgical risks [9–12]. The MRI component ensures accurate targeting (spatial resolution of 1 mm) and continuous temperature monitoring (accuracy of 1 °C) for safety [13–16]. MRgFUS’s advantages include immediate effects compared to radiotherapy’s delayed response, repeatability for recurrent pain, and the ability to treat multiple sites in a single session [17, 18]. ‌

To date, the existing expert consensus was generated in 2015 by a team of 14 international experts at the European Symposium on Focused Ultrasound Therapy. As this was developed against the backdrop of completing the Phase III clinical trial of MRgFUS for painful bone metastases, the consensus leaned toward the technical principles and future development directions of MRgFUS. ‌ Over the subsequent decade, MRgFUS has been progressively adopted in clinical practice for painful bone metastases across regions, creating an urgent need for standardized operational protocols. To address this gap, 29 experts from relevant fields in China convened a structured face-to-face consensus meeting, conducting extensive topic-specific deliberations and anonymous voting throughout the session. Ultimately, the meeting developed a comprehensive surgical workflow alongside critical clinical considerations for MRgFUS application. This consensus provided standardized implementation recommendations for MRgFUS in ‌the treatment of painful bone metastases, covering patient selection criteria, procedural protocols, efficacy evaluation methods, and safety management. These recommendations aim to establish best practices for this application, with a focus on technical considerations and quality control measures.

## Methodology

### Expert panel composition for the consensus

Consensus formation in this study was achieved through a structured face-to-face consensus meeting. The expert panel for this consensus was selected via multicenter collaboration, encompassing representative hospitals across multiple provinces in China, and involving disciplines closely tied to the clinical and interventional management of metastatic bone tumors (including Radiology, Orthopedics, Interventional Radiology, and Oncology). All participating experts possess at least 5 years of professional experience in the clinical diagnosis, treatment, or interventional management of metastatic bone tumors (backed by extensive clinical practice and specialized disciplinary backgrounds). A total of 29 eligible experts were included in the development of this consensus. Detailed information about this expert panel, which covers the specific hospitals, corresponding departments, and the number of participating experts, is provided in Table S1.

### Systematic literature review

Prior to the consensus meeting, a steering committee was formed consisting of 7 multidisciplinary experts nominated by the Radiology Department Association of Hospitals of Shanghai and the Micro/Non-invasive Treatment Committee of the Chinese Research Hospital Association, which conducted a systematic literature review. The specific names, disciplines, and professional experience durations of these committee members have been listed in Table S2. Relevant literature was retrieved from PubMed, Embase, and the Cochrane Collaboration Library to identify publications on MRgFUS for the treatment of painful bone metastases published between January 2000 and June 2025, with the overall search strategy utilizing MeSH terms as well as terms related to MRgFUS and bone metastases (detailed search terms are available in Table S3). The inclusion criteria were as follows: (1) randomized controlled trials, prospective cohort studies, retrospective cohort studies, systematic reviews/meta-analyses, and case series with high methodological quality (≥ 10 patients); (2) MRgFUS used as the primary or palliative treatment for painful bone metastases; (3) presence of specific outcome measures such as pain relief (e.g., NRS/BPI-QoL scores) and treatment safety (adverse events graded by CTCAE); (4) peer-reviewed journal articles or registered clinical trial reports (excluding conference paper and gray literature with unavailable extractable data). The exclusion criteria were: (1) studies involving non-painful bone metastases, primary bone tumors, benign conditions, or hyperthermia; (2) interventions combining MRgFUS with other treatment technologies (e.g., radiofrequency ablation) without separate outcome data; (3) animal experiments, basic research, or in vitro studies. Based on the included literature and the opinions of the expert panel, initial statements were drafted covering the following aspects: (1) indications, (2) contraindications, (3) preoperative evaluation, (4) intraoperative procedures, and (5) postoperative follow-up. After the completion of the initial consensus draft, it was disseminated online to the remaining 22 panel experts for preliminary review ahead of the formal consensus meeting.

### Consensus formulation

At the 2025 joint conference hosted by the Radiology Department Association of Hospitals of Shanghai and the Micro/Non-invasive Treatment Committee of the Chinese Research Hospital Association, all 29 panel experts convened to review the initial statements and put forward individual feedback on the draft. Subsequently, experts conducted in-depth, topic-by-topic deliberations to refine the content in real time. Following revisions, anonymous voting was conducted for each finalized statement using an electronic polling system to ensure impartiality and confidentiality in the decision-making process. Votes were categorized into three predefined levels: A (strong consensus: ≥ 95% agreement), B (consensus: 75–94% agreement), and C (majority agreement: 50–74% agreement). For any statement initially scoring near the B-level threshold, experts re-discussed key contentious points to refine wording and evidence support before a second vote. Ultimately, all statements achieved either Level A or Level B, with no statements falling into Level C (Table 1).

**Table 1:** Expert consensus recommendations on MRgFUS for painful bone metastasis management

Items	Consensus statements	Category and votes*
Indications	• Bone metastases with persistent pain (NRS ≥ 4) • Accessible sites: ribs/sternum/limbs/pelvis/scapulae/posterior portion of vertebrae below L3 • Overlying soft tissue ≥ 1 cm • No fracture risk • MRI visible • Unobstructed acoustic pathway	A (28/29)
Contraindications	• Unstable pathological fractures (displacement risk) • > 5 bone lesions • Recent radiotherapy/chemotherapy (within 4 weeks) • Life expectancy < 3 months • Active cardiovascular disease/infection/coagulopathy • MRI or anesthesia contraindications	B (27/29)
Preprocedural		
Patient evaluation	• 3-stage MRgFUS evaluation: technical suitability, superior risk-benefit vs. alternatives, and consensus enrollment. • NRS + BPI-QoL pain assessment and MEDD calculation	A (29/29)
Patient preparation	Routine tests (complete blood count, electrocardiogram, etc.), informed consent, skin and bowel preparation, and anesthesia evaluation.	A (29/29)
MRI assessment	T1WI for baseline measurement and T2WI for component differentiation. All sequences spatially aligned for surgical planning.	A (29/29)
Intraprocedural		
Team collaboration	Multidisciplinary team (physician, MRI technician, anesthesiologist, nurse, assistant) collaborates for real-time guidance, sedation, and monitoring.	A (29/29)
Target ablation	Low-energy ultrasound verifies lesion location with subsequent acoustic power adjustment to achieve 65–85 °C at the bone interface.	A (29/29)
Real-time thermal monitoring	PRF-based MRI monitors real-time thermometry. T1WI-CE immediately post-sonication assesses treatment efficacy via NPV calculation.	A (28/29)
Postprocedural		
Care protocol	30-min supine positioning with hemodynamic monitoring, and local treatment area assessment (erythema/edema/tenderness).	A (29/29)
Long-term management	Structured follow-ups at 1/3 days, 1/2 weeks, and 1/2/3/6 months with imaging (CT/MRI) and pain scales (NRS/BPI-QoL/MEDD).	A (28/29)

*NRS* numeric rating scales, *BPI-QoL* Brief Pain Inventory–Quality of Life, *MEDD* morphine equivalent daily dose, *PRF* proton resonance frequency, *NPV* non-perfused volume

* A (strong consensus: ≥ 95%), B (consensus: 75–94%), and C (majority agreement: 50–74%)

## Indications and contraindications of MRgFUS for painful bone metastases

MRgFUS may be considered for pain palliation in bone metastases when the following criteria are met: (1) Patients with confirmed bone metastases causing persistent pain (Numerical Rating Scale (NRS) ≥ 4) despite standard therapies. (2) The bone metastatic lesion is located in accessible anatomical sites, including ribs, sternum, limb bones, pelvic bones, scapulae, or the posterior portion of vertebrae below L3 (some experts suggest L2 may be feasible without neurological injury). (3) Cases where radiotherapy is contraindicated, ineffective, or refused by the patient. As radiotherapy effects ‌typically emerge‌ within 4 weeks [19], the criteria for declaring radiotherapy ineffective are: patients report no alleviation of self-reported pain and no reduction in analgesic consumption within 4 weeks, or experience recurrence of pain. (4) Metastatic lesions with intact overlying soft tissue (≥ 1 cm thickness for safe acoustic transmission [20]). (5) Structurally stable bones with no evidence of impending fracture. (6) The lesion should be sufficiently visible on pre-contrast MR images. (7) The target lesion must lie within an unobstructed acoustic pathway, free from significant scar tissue, gas-filled organs, or overlying bone.

The following conditions are considered contraindications: (1) Unstable pathological fractures are susceptible to displacement or exacerbation during sonication. (2) More than five painful bone lesions were observed (micrometastases without bone destruction excluded, e.g., prostate cancer-related). (3) Patients receiving radiotherapy or chemotherapy for pain management within the preceding 4 weeks. (4) Patients with a life expectancy under 3 months derive minimal clinical benefit. (5) Patients exhibited uncontrolled cardiovascular disease (‌New York Heart Association (NYHA) Class III/IV heart failure, acute coronary syndrome, or ventricular arrhythmias), active infection (e.g., sepsis, osteomyelitis), or coagulopathy (e.g., ‌international normalized ratio (INR) > 1.5) [21–24]. (6) MRI contraindications include ‌absolute contraindications‌ such as metallic implants (e.g., non-MRI-compatible pacemakers) and gadolinium allergy, and ‌relative contraindications‌ such as severe claustrophobia [25]. (7) General/epidural anesthesia or general sedation cannot be administered to patients due to reasons such as drug allergies or intolerance to prolonged immobilization [26].

## The preoperative comprehensive evaluation for patients

**Fig. 1:**
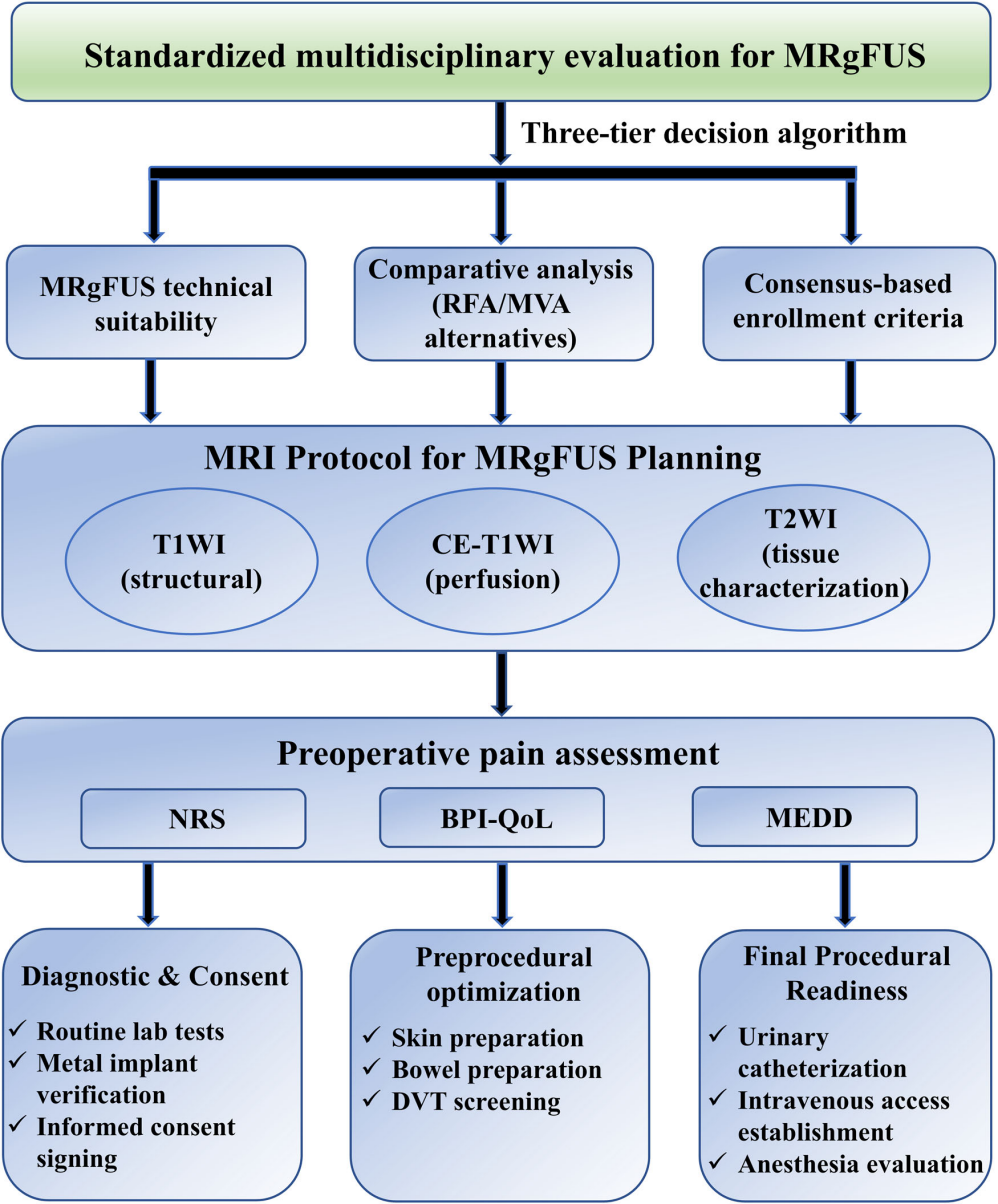
Preoperative operational workflow for MRgFUS treatment of painful bone metastases

The MRgFUS preoperative evaluation protocol requires standardized multidisciplinary review by a tumor board, including diagnostic radiologists (tumor characterization), interventional radiologists (procedural feasibility), orthopedic oncologists (skeletal stability), and medical/surgical oncologists (systemic disease control). This process employs a three-tier algorithm: (1) technical suitability assessment for MRgFUS, (2) comparative analysis with alternative modalities, and (3) final consensus-based enrollment decision. Patients are enrolled only upon confirming: technical suitability for ultrasound ablation, superior risk-benefit ratio over other minimally invasive options, and absence of contraindications through collegial review (Fig. 1). Regarding the comparative selection of alternative therapies, radiofrequency ablation (RFA) and microwave ablation (MVA)‌ provide precise tumor necrosis with rapid pain relief (1–3 days), particularly effective for small lesions (< 3 cm) but limited by proximity to critical structures [27]. ‌Cementoplasty‌ excels in mechanical stabilization of osteolytic lesions, achieving immediate pain reduction (24–48 h) and fracture prevention, though its efficacy diminishes in extensive soft tissue involvement [8]. Radiotherapy‌ remains the gold standard for diffuse metastases, with pain relief typically delayed (2–4 weeks) but durable [19]. ‌MRgFUS‌ stands out as a non-ionizing, incision-free option, demonstrating rapid pain relief, yet its accessibility is constrained by cost and technical complexity. Ultimately, the choice hinges on lesion characteristics (size/location) and patient performance status.

The preoperative MRI protocol employs T1-weighted imaging (T1WI) for anatomical delineation of bone structures and identifying tumor infiltration (typically hypointense against hyperintense fatty marrow). T1WI also assists in assessing cortical integrity and identifying sclerotic components that may affect ultrasound penetration. ‌Enhanced T1WI was performed immediately after surgery and compared with preoperative T1WI to calculate the non-perfused volume (NPV) ratio, which is calculated as the percentage of non-enhanced tumor volume relative to the original tumor volume [28]. This parameter serves as a critical intraprocedural biomarker for predicting histological necrosis extent. A multicenter clinical study using binary logistic regression found that the NPV ratio could effectively predict mid-term MRgFUS treatment outcomes [26, 29]. T2-weighted imaging (T2WI) supplements the assessment by highlighting bone marrow edema and soft tissue involvement, as well as differentiating solid tumor components from cystic/necrotic areas, thereby aiding in accurate targeting and treatment planning. Notably, within months after radiotherapy, both bone marrow and soft tissue may exhibit high signal intensity on T2WI, reflecting post-treatment inflammation. The specific imaging parameters for T1WI and T2WI are shown in ‌Table S4‌. All sequences are spatially aligned‌ to assess osseous ultrasound interference zones and establish safety buffers around critical neural structures. Multiplanar reformats are then generated from the aligned volumetric data, enabling optimized spatial relationship analysis for surgical planning.

Preoperative pain assessment begins with documenting baseline pain intensity using the NRS (0–10), where 0 indicates no pain and 10 represents the worst imaginable pain, along with detailed pain characteristics including location, quality, duration, and aggravating/relieving factors. The Brief Pain Inventory–Quality of Life (BPI-QoL) measured pain severity by evaluating interference across seven domains (activity, mood, walking, work, relationships, sleep, and life enjoyment) on a 0–10 scale (0 = no interference, 10 = complete interference). For patients on opioid therapy, the Morphine Equivalent Daily Dose (MEDD) is calculated to quantify opioid exposure, with particular attention to those meeting opioid-tolerant criteria (typically ≥ 60 mg MEDD for ≥ 1 week). The medication history should comprehensively record all analgesics (NSAIDs, opioids, and adjuvants—specifying names, dosages, frequency, and efficacy), prior adverse drug reactions, and patient-reported pain control satisfaction. Preoperative fracture risk assessment should be performed using validated tools such as the Mirels’ Classification system (Mirel score ≤ 7) [30, 31]. This integrated evaluation informs personalized perioperative analgesia strategies, ensuring appropriate dose adjustments for opioid-tolerant patients while mitigating risks of inadequate pain relief.

The patient’s preoperative preparation includes: completing routine tests such as complete blood count, urinalysis, stool test, coagulation profile, electrocardiogram‌, tumor markers; thoroughly explaining to the patient the benefits, limitations, expected outcomes, potential complications, and side effects of MRgFUS therapy while understanding their expectations; verifying the absence of metal implants near the treatment area; obtaining signed informed consent from the patient; shaving and cleaning the skin overlying the target bone; recommending 8 h of liquid diet and bowel preparation; performing lower extremity venous ultrasound for patients at high risk of deep vein thrombosis (DVT); inserting a urinary catheter and establishing intravenous access 30 min pre-procedure; and conducting anesthesia evaluation (with anesthesiologist involvement).

## MRgFUS surgical procedure protocol

### MRgFUS team roles and collaborative workflow

The MRgFUS team typically consists of multiple specialized roles working collaboratively: at least one doctor (typically a radiologist or an interventional radiologist), one assistant, one MRI technician, one nurse, and one anesthesiologist. The doctor serves as the primary operator, directing the therapeutic protocol and making real-time treatment decisions while overseeing image interpretation and target lesion identification. The MRI technician ensures optimal MRI sequences and system calibration, while also assisting with patient positioning and coil setup. The anesthesiologist administers sedation or analgesia as needed, and the nurse monitors vital signs and manages intravenous access. The assistant supports the team by handling data recording and equipment troubleshooting.

### Technical specifications of MRgFUS

This expert consensus uses the ExAblate 2000 system (InSightec Ltd.) as an example, as it is one of the most clinically established MRgFUS platforms for noninvasive thermal ablation treatments and remains the main system with FDA premarket approval [32]. Additionally, the parameters and technical considerations covered in this expert consensus are applicable to other MRgFUS platforms as well. The MRgFUS procedure begins with acquiring diagnostic MR images of the target area using the integrated 1.5-T or 3.0-T MRI scanner. The doctor reviews these images on the system workstation to identify the target volume, delineate treatment contours, and finalize the treatment plan, with therapy planning software automatically calculating optimal treatment parameters. The system features a 208-element phased array ultrasound transducer with a 120 mm diameter, capable of delivering focused ultrasound beams at depths ranging from 60 to 200 mm. The transducer operates within an acoustic frequency window of 0.95–1.3 MHz and delivers energy ranging from 200 to 7200 J per sonication. While high-frequency ultrasound limits penetration depth, low-frequency ultrasound accesses deeper tissues. Research indicates that frequencies around 1 MHz are most effective for optimal thermal deposition [33]. The transducer employs a wide-beam technique, positioning the focal point posterior to the bone cortex while placing the ablation zone at the intersection between the ultrasound beam and the bone surface. This configuration allows precise thermal ablation under real-time MRI guidance, with the MR system providing both anatomical imaging and temperature mapping during treatment.

### Intraoperative MRgFUS protocol

The MRgFUS treatment protocol requires patients to receive intravenous unconscious sedation/analgesia with fentanyl citrate (2 mcg/kg) and dexmedetomidine hydrochloride (1 mcg/kg). Regional anesthesia may be adjunctively administered when substantial postoperative pain is anticipated [34]. During sonication, the ultrasound beam must maintain a minimum 1 cm clearance from critical functional organs and neurovascular bundles, with absolute contraindications for skull, cervical vertebrae, thoracic vertebrae, and L1 lumbar vertebrae lesions to prevent CNS injury [35]. All potential sources of ultrasonic aberration (including surgical scars, metal implants, subcutaneous air pockets, bowels or calcifications) must be identified on pretreatment MRI and avoided during beam path planning [32]. Strategic inter-sonication intervals are essential to prevent microbubble generation. Such bubbles may scatter and disrupt the acoustic field, leading to incomplete target ablation and adjacent tissue damage [36]. For patients with multiple eligible bone metastases, sequential treatment of all sonication-accessible lesions is permitted. Continuous intraoperative monitoring includes real-time MR thermometry coupled with physiological surveillance, with automatic beam termination triggered by either system-detected anomalies or manual activation via the patient-controlled emergency stop button positioned within hand’s reach. This dual safety mechanism ensures immediate cessation of energy delivery upon either physiological parameter deviation or patient-reported discomfort.

### Real-time thermal monitoring and control in MRgFUS

**Fig. 2:**
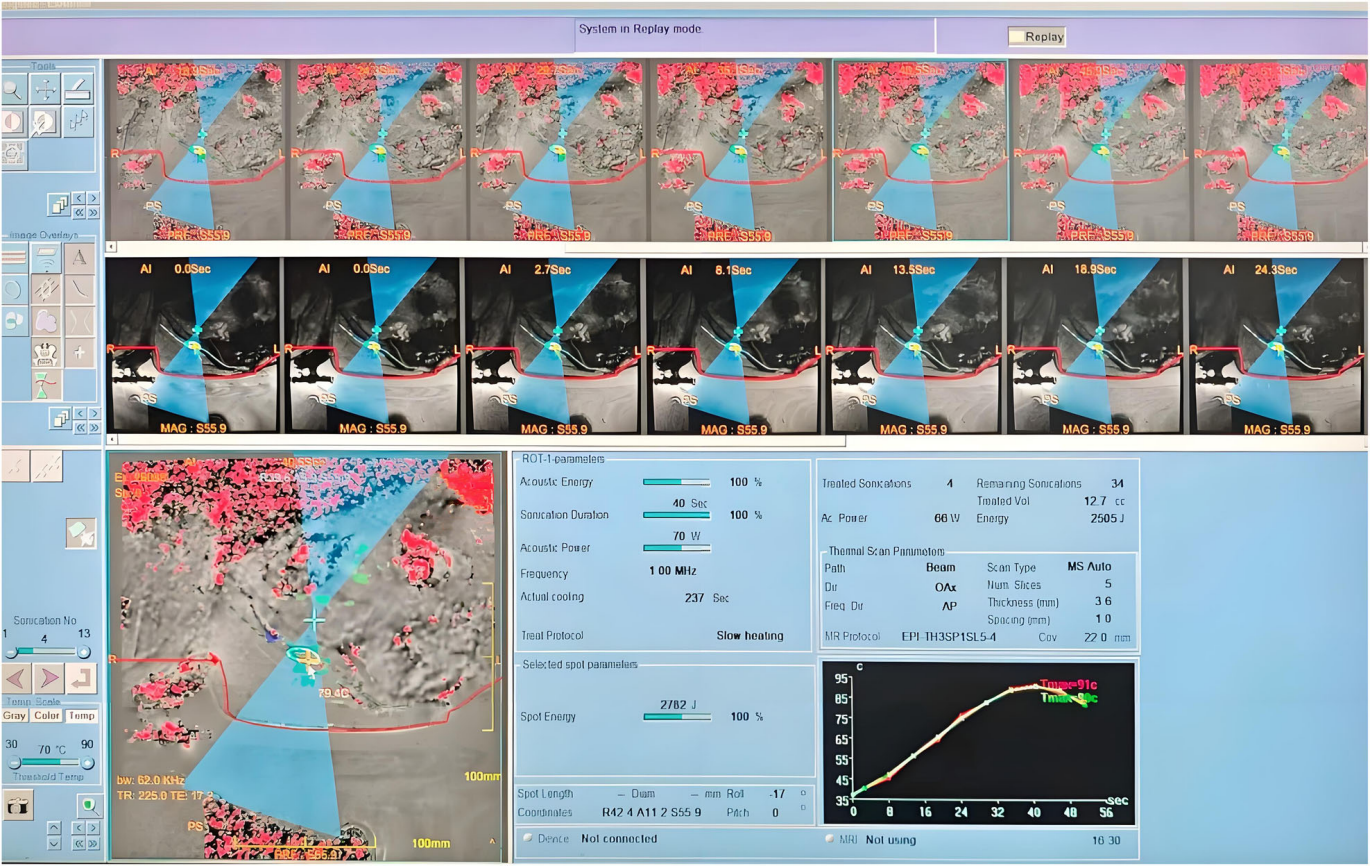
The actual working interface for temperature measurement during MRgFUS. This is a male patient with iliac bone metastasis, where the red line represents the skin surface, the white line indicates the bone cortex interface, the blue beam signifies the path of the ultrasound beam, the green circle denotes the actual treatment area, the yellow cursor marks the location for temperature measurement, and the curve in the bottom right corner represents real-time temperature changes in the monitored region

The intraoperative temperature control during MRgFUS treatment employs a sophisticated real-time feedback system. Based on pretreatment planning that calculates tumor volume and required sonication spots (typically 25 s per spot), the procedure initiates with low-energy verification sonications to confirm focal positioning. The temperature measurement sequence of MRgFUS is based on Echo Planar Imaging (EPI), with a typical spatial resolution of 1944 × 1944 µm and a time resolution of 126 ms. During therapeutic sonication, continuous MRI thermometry utilizing proton resonance frequency (PRF) shift methodology generates quantitative temperature maps across the treatment field, displayed as both spatial heat distribution and temporal thermal curves [37]. The schematic diagram of the actual operation for temperature measurement is shown in Fig. 2. The system dynamically modulates three key parameters: (1) Acoustic power (adjusted to maintain 65–85 °C at the bone-tissue interface), (2) Spot geometry (optimized based on real-time thermal spread patterns), and (3) Sonication frequency (tuned for optimal energy deposition). Each thermal dose is maintained for ≥ 5 s to ensure irreversible coagulation necrosis. For cortical bone metastases, temperature monitoring focuses on periosteal surfaces, whereas lytic lesions require tracking through cortical defects. Recent clinical evidence demonstrates that increased energy density delivered to the osseous surface correlates with improved analgesic outcomes [38]. The thermal feedback loop continues until complete volumetric coverage is achieved, with post-treatment T1/T2-weighted MRI immediately verifying ablation margins.

### Postprocedural care protocol

Postoperative care for MRgFUS-treated bone tumor patients involves immediate removal of urinary catheters and intravenous access, followed by comprehensive monitoring, including vital signs assessment and focused evaluation of the treatment area for erythema, edema, or tenderness. Patients maintain supine positioning for 30 min with continuous hemodynamic surveillance while emphasizing expected transient bone pain (managed with ice packs), NSAIDs avoidance for 72 h to prevent interference with bone healing evaluation.

## Postprocedural adverse events

The common adverse events after treatment are shown in Table 2. The patient’s adverse reactions should be classified according to the Common Terminology Criteria for Adverse Events (CTCAE) version 6.0, with MRgFUS treatment-related adverse events typically graded into three levels: (Grade 1: Mild; Grade 2: Moderate; Grade 3: Severe) [19]. Hurwitz et al and Lee et al reported that the most common adverse reaction was pain during the MRgFUS procedure, with the incidence rates of 32.1% and 33%, respectively [19, 39]. A recent meta-analysis of 26 studies involving 799 patients reported a severe adverse event rate of only 0.9%, including 1 case of DVT, 2 skin burns, and 4 cases of fractures [7]. A four-center clinical study by Yin et al demonstrated that mild adverse events occurred in 4.2% of cases, while severe adverse events were observed in 2.1%, and most events were self-limiting or manageable with conservative measures within 52 days, indicating that MRgFUS is a relatively safe procedure [29]. In the studies by Yin et al and Hurwitz et al, all grade III adverse events consisted of skin ulcers/burns and fractures. Skin complications occurring due to insufficient tumor-to-skin distance necessitate strict adherence to the ≥ 1 cm inclusion criterion, while fractures were associated with treatment of weight-bearing bones or osteoporotic conditions, requiring thorough preoperative risk assessment and postprocedural skeletal protection measures [29, 39].

**Table 2:** Common adverse events following MRgFUS treatment

Category	Specific events
General disorders and administration site conditions	Fever Fatigue Dry mouth Hemorrhage Localized edema Pain during and after treatment
Musculoskeletal and connective tissue disorders	Bone fracture Myositis
Digestive system	Diarrhea Anorexia Constipation Nausea
Nervous system disorders	Nerve injury and dysesthesia
Skin and subcutaneous tissue disorders	Skin burn and ulceration Hypohidrosis Dermatitis
Vascular disorders	Deep venous thrombosis

## Postprocedural assessment and longitudinal follow-up

Postoperative evaluation and follow-up are being conducted by a multidisciplinary team including senior and junior physicians along with specialized nurses. Standardized follow-ups are conducted at protocol-defined intervals (1/3 days, 1/2 weeks, and 1/2/3/6 months), incorporating clinical examinations and comprehensive pain assessment through NRS, BPI-QoL, and MEDD tracking. Postoperative CT/MRI (with or without contrast enhancement) should be performed at 3 months, and tumor response is evaluated based on the MD Anderson Cancer Center’s bone metastasis-specific criteria (MDA criteria), focusing on size changes and sclerotic filling of the treated lesions [40, 41]. Treatment response is being categorized as complete responder (CR: NRS = 0 with ≤ 25% MEDD increase), effective responder (ER: NRS reduction ≥ 2 points with ≤ 25% MEDD increase), or non-responder (NR), with all adverse events being systematically documented throughout the 6-month observation period. In a three-phase randomized trial enrolling 147 participants, the MRgFUS group demonstrated a clinically meaningful response rate of 64.3% for the primary endpoint, significantly outperforming the placebo group (20.0%) (*p* < 0.001) [39]. ‌The clinical study by Gu et al demonstrated significant pain reduction in patients with recurrent or refractory pain after radiotherapy/chemotherapy [42]. The NRS scores decreased from 6.0 ± 1.5 at baseline to 3.7 ± 1.7 at 1-week post-treatment, further declining to 3.1 ± 2.0 at 1-month and 2.2 ± 1.0 at 3-month follow-up (all *p* < 0.01). In a matched-pair clinical study by Lee et al, MRgFUS demonstrated superior therapeutic efficacy compared to conventional radiotherapy in 63 patients with painful bone metastasis. Notably, MRgFUS exhibited significantly faster pain palliation, achieving a markedly higher response rate at 1-week post-treatment (71% vs. 26%, *p* = 0.0009) [19]. A meta-analysis by Baal et al, encompassing 33 studies (1082 patients), reported a 79% (95% CI: 73–83%) complete or effective response rate at 3-month follow-up in patients with painful bone metastases treated with MRgFUS [7]. According to a recent multicenter clinical study (*N* = 82), the composite rate of CR and ER reached 75.6% (19.5% CR + 56.1% ER) at 3-month follow-up, demonstrating comparable efficacy to the aforementioned results [29]. This suggests that MRgFUS is an effective treatment for bone pain, offering advantages such as rapid onset and long-lasting effects.

### Factors related to the therapeutic efficacy of MRgFUS

To date, a considerable number of clinical studies have validated factors related to the therapeutic efficacy of MRgFUS for painful bone metastases. First, a study indicates that a patient’s pretreatment NRS score and their Karnofsky Performance Status (KPS) scores (a scale assessing physical functional capacity and self-care ability) significantly impact treatment efficacy. Specifically, a higher pretreatment NRS level combined with a lower KPS score predicts poorer therapeutic outcomes [40]. Another study has demonstrated that the energy density delivered by focused ultrasound to the bone surface and postoperative periosteal vessel detachment (formation of a ‘black band’) increases the likelihood of bone pain in patients [38]. However, it should be noted that excessive ultrasonic energy may pose a risk of peripheral tissue injury. Furthermore, osteoblastic bone metastasis lesions exhibit superior therapeutic efficacy compared to osteolytic lesions. This is attributed to the fact that osteoblastic lesions, through the formation of bone surfaces, reflect more focused ultrasound waves onto periosteal nerves. In contrast, osteolytic lesions induce pain exacerbation via acidic tumor microenvironments that sensitize afferent nerves [29]. Notably, studies have collectively demonstrated that tumor size bears no significant correlation with pain relief, which is attributable to the fact that the therapeutic mechanism of MRgFUS lies in the ablation of periosteal nerves rather than the tumor itself [29, 40].

### Limitations of MRgFUS in treating painful bone metastases

Despite demonstrating satisfactory efficacy and safety in clinical trials, MRgFUS therapy faces practical challenges in real-world clinical practice. First, the reproducibility of MRgFUS may vary across centers due to differences in operator experience and equipment calibration. The operator needs to accurately identify the lesion area through MRI images and focus ultrasound energy on the target location. Any small positioning deviation can lead to inadequate or excessive treatment, thereby affecting efficacy or increasing the risk of complications. Therefore, the operator needs to undergo rigorous training from experienced professionals and engage in detailed discussions with the operating team to develop a treatment plan. Second, the high cost of MRgFUS therapy remains a significant barrier to its widespread adoption. In China, MRgFUS for painful bone metastases typically costs between ‌¥25,000 and 30,000 and is ‌not covered by national health insurance. In countries like the United States, MRgFUS has been reimbursed by both government programs and private payers [18]. Third, the limitations also include the requirement for ‌MR-compatible HIFU units‌ [43]. This specialized equipment is costly and not widely available in medical facilities. Additionally, the need for MRI compatibility imposes technical constraints on treatment protocols, such as avoiding metallic components in the device. Furthermore, MRgFUS for painful bone metastases has a ‌limited application scope‌, primarily targeting ‌appendicular bones (limbs)‌ and ‌peripheral skeletal sites‌. Notably‌, the lesion location is critical for MRgFUS implementation, requiring at least 1 cm distance from skin and neurovascular bundles to avoid adverse events and damage to critical structures, while the ultrasound path must also avoid intestinal structures to prevent energy scattering and bowel wall injury. This anatomical restriction reduces its clinical utility for a significant proportion of patients.

## Future perspectives

MRgFUS ablation as a groundbreaking therapeutic advancement for pain palliation and tumor control in bone metastasis patients, particularly serving as an alternative therapy for radiation-resistant cases (including patients with inadequate symptom relief post-radiation, contraindications to further external beam radiation therapy, or preference for noninvasive options) [44]. This modality’s superior safety profile, characterized by negligible adverse events, positions it as a compelling alternative to conventional palliative treatments. Leporace et al demonstrated significantly higher local tumor control rates in bone metastases treated with MRgFUS (84%) compared to percutaneous thermal ablation techniques (65%) [45]. The mechanism involves precise acoustic energy deposition on cortical bone surfaces, generating localized hyperthermia that induces targeted neurolysis of the highly innervated periosteum—demonstrating exceptional efficacy in pain modulation. Simultaneously, the accumulated thermal energy within metastatic soft tissue promotes controlled necrosis, which may contribute to secondary benefits including tumor volume reduction, medullary bone remineralization, and potential disease-modifying effects. Therefore, MRgFUS exerts dual therapeutic effects: periosteal denervation mediates analgesia, while tumoral thermoablation achieves cytoreduction. These mechanisms necessitate global multicenter registry studies to standardize protocols, refine energy parameters, and concurrently assess long-term survival and patient-reported outcomes.

In the future, AI-driven precision medicine shows significant promise in advancing MRgFUS for painful bone metastases. Deep learning algorithms will analyze comprehensive datasets, including pretreatment MRI characteristics, treatment parameters, pain relief outcomes, and adverse events, to create predictive models for patient selection. By processing multimodal imaging data through convolutional neural networks, these systems will identify optimal candidates based on tumor location, cortical integrity, and periosteal involvement patterns, significantly improving treatment predictability. Furthermore, AI-powered treatment planning systems will autonomously optimize sonication pathways using 3D anatomical reconstructions, calculating ideal energy deposition patterns while avoiding critical structures through real-time thermal dose simulations. Reinforcement learning models will continuously refine ablation parameters (intensity, duration, focal points) based on outcomes from global treatment registries. AI integration will standardize MRgFUS procedures by optimizing efficiency and therapeutic precision, simultaneously reducing operator dependency through automated data-driven personalization. This advancement will ultimately position MRgFUS as a standardized and widely adopted image-guided ablation modality in metastatic bone disease management.

## Conclusion

MRgFUS effectively relieves pain in bone metastasis patients by thermally ablating periosteal nerve endings. Currently positioned as a second-line palliative treatment, MRgFUS demonstrates clinical advantages over conventional therapies by delivering faster pain relief, causing fewer side effects, and exhibiting better patient tolerance. The therapeutic potential of MRgFUS can be amplified by leveraging worldwide clinical data aggregation and AI-driven protocol refinement, which would not only establish standardized treatment processes but also enhance therapeutic efficiency, thereby facilitating broader clinical implementation for painful bone metastases.

## Supplementary information

Supplementary information

## Abbreviations

BPI-QoL: Brief Pain Inventory–Quality of Life
CR: Complete responder
DVT: Deep vein thrombosis
ER: Effective responder
MEDD: Morphine equivalent daily dose
MRgFUS: Magnetic resonance-guided focused ultrasound surgery
NPV: Non-perfused volume
NRS: Numerical Rating Scale
NSAIDs: Nonsteroidal anti-inflammatory drugs
PRF: Proton resonance frequency
