# Supplementary material for: Chinese expert consensus on magnetic resonance-guided focused ultrasound surgery for painful bone metastases
Source: Insights Imaging. 2026 Apr 7;17:89. doi: 10.1186/s13244-026-02268-7 (PMC13057040; doi:10.1186/s13244-026-02268-7)
Supplement: Supplementary file 1 — Supplementary information [file 13244_2026_2268_MOESM1_ESM.pdf]

Chinese expert consensus on magnetic resonance-guided focused ultrasound surgery for painful bone metastases

ELECTRONIC SUPPLEMENTARY MATERIAL

Table S1 The panel of experts who participated in developing the consensus.

Province	Hospital	Department	Number
Shanghai	[1]Shanghai General Hospital	Radiology	11
	[2]Ruijin Hospital	Orthopedics	5
	[3]Shanghai Tenth People's Hospital	Interventional Radiology	3
	[4]Shanghai Cancer Center	Oncology	2
	[5]Huashan Hospital		
	[6]Shanghai Changhai Hospital		
Jiangsu	[7]Affiliated Hospital of Nantong	Oncology	2
	University	Interventional Radiology	1
	[8]Jiangsu Province Hospital		
Beijing	[9]PLA Air Force Medical Center	Interventional Radiology	1
Hubei	[10]Union Hospital, Tongji Medical	Interventional Radiology	1
	College		
Shaanxi	[11]Tangdu Hospital	Radiology	1
Henan	[12]Henan Provincial People's Hospital	Radiology	1
Guangdong	[13]Foshan Hospital of TCM	Radiology	1

PLA: People’s Liberation Army; TCM: Traditional Chinese Medicine

Table S2 Composition of the consensus steering committee.

Name	Hospital	Department	Professional experience
Han Wang	Shanghai General Hospital	Interventional Radiology	25 years
Wen Wang	Tangdu Hospital	Radiology	28 years
Tang Na	Shanghai General Hospital	Radiology	25 years
Jiakang Shen	Shanghai General Hospital	Orthopedics	12 years
Junhai Zhang	Huashan Hospital	Radiology	28 years
Shengping Wang	Shanghai Cancer Center	Radiology	28 years
Qiang Xue	Affiliated Hospital of Nantong University	Oncology	28 years

Table S3 Search strategies for MRgFUS in painful bone metastasis across databases.

Database	Search strategy
PubMed	(“High-Intensity Focused Ultrasound Ablation”[Mesh] or “HIFU” or “Magnetic resonance guided focused ultrasound” or “MRgFUS”) and (“Bone Neoplasms”[Mesh] or “Bone metastases”)
Embase	(“Bone metastasis”/exp or “Bone Neoplasms”/exp) AND (“Magnetic resonance guided high intensity focused ultrasound”/exp or “MRgFUS”/exp or “High-Intensity Focused Ultrasound”/exp or “HIFU”/exp)
Cochrane Collaboration Library	(Bone or Osseous or Skeletal) AND (Magnetic resonance guided high intensity focused ultrasound or MRgFUS or High-Intensity Focused Ultrasound or HIFU)

Table S4 Preoperative axial MRI imaging protocol.

MRI Acquisition Parameters	T1WI	T2WI
Series Description	SE	SE
Repetition time/echo time (TR/TE) (ms)	472/12.1	11861.9/100.6
Pixel bandwidth (KHz)	81.3	488.2
Slice thickness (mm)	7	6
Spacing between slices (mm)	9	8
Frequency	127.7	127.7
Echo train length	3	28
Flip angle	111°	160°
